# An Accurate and Efficient Approach to Knowledge Extraction from Scientific Publications Using Structured Ontology Models, Graph Neural Networks, and Large Language Models

**DOI:** 10.3390/ijms252111811

**Published:** 2024-11-03

**Authors:** Timofey V. Ivanisenko, Pavel S. Demenkov, Vladimir A. Ivanisenko

**Affiliations:** 1 The Artificial Intelligence Research Center of Novosibirsk State University, Pirogova Street 1, Novosibirsk 630090, Russia; demps@bionet.nsc.ru (P.S.D.); salix@bionet.nsc.ru (V.A.I.); 2 Institute of Cytology & Genetics, Siberian Branch, Russian Academy of Sciences, Prospekt Lavrentyeva 10, Novosibirsk 630090, Russia

**Keywords:** text-mining, ANDSystem, deep learning, GNN, LLM, knowledge graph

## Abstract

The rapid growth of biomedical literature makes it challenging for researchers to stay current. Integrating knowledge from various sources is crucial for studying complex biological systems. Traditional text-mining methods often have limited accuracy because they don’t capture semantic and contextual nuances. Deep-learning models can be computationally expensive and typically have low interpretability, though efforts in explainable AI aim to mitigate this. Furthermore, transformer-based models have a tendency to produce false or made-up information—a problem known as hallucination—which is especially prevalent in large language models (LLMs). This study proposes a hybrid approach combining text-mining techniques with graph neural networks (GNNs) and fine-tuned large language models (LLMs) to extend biomedical knowledge graphs and interpret predicted edges based on published literature. An LLM is used to validate predictions and provide explanations. Evaluated on a corpus of experimentally confirmed protein interactions, the approach achieved a Matthews correlation coefficient (MCC) of 0.772. Applied to insomnia, the approach identified 25 interactions between 32 human proteins absent in known knowledge bases, including regulatory interactions between MAOA and 5-HT2C, binding between ADAM22 and 14-3-3 proteins, which is implicated in neurological diseases, and a circadian regulatory loop involving RORB and NR1D1. The hybrid GNN-LLM method analyzes biomedical literature efficiency to uncover potential molecular interactions for complex disorders. It can accelerate therapeutic target discovery by focusing expert verification on the most relevant automatically extracted information.

## 1. Introduction

The volume of scientific literature is increasing rapidly, with an average annual growth of 4.10% and a doubling time of 17.3 years [1]. The PubMed database alone contains over 37 million abstracts on biomedical topics, with approximately 1.5 million documents added annually. Such quantities of published literature make it challenging for researchers to stay up-to-date with the most relevant information. Studying complex biological systems, in particular, is impossible without integrating knowledge from a variety of sources, such as scientific literature, databases, and experimental data [2]. One way to address this problem is by applying automated text analysis methods [3], which can be divided into traditional and artificial intelligence (AI) approaches.

### 1.1. Traditional Text-Mining Approaches in Biomedical Research

Many text-mining methods mainly focus on one of two natural language processing tasks, named entity recognition and relation extraction [4,5]. Named entity recognition identifies and categorizes key biomedical entities such as genes, proteins, diseases, and drugs within the text [6]. A well-known class of approaches to named entity recognition is the dictionary-based methods, which rely on comprehensive dictionaries to map terms found in text to known entities. This allows for linking entities with external curated databases, grouping synonyms, and achieving high precision. Previously, we used it in the computational tool ANDSystem [7,8], designed for the automated extraction of structured biological knowledge from the text of scientific publications, databases, and patents, representing it in the form of associative gene networks (AGNs). This approach was also employed in the ANDDigest module of ANDSystem [9,10], intended for searching documents in PubMed based on the predefined ontology of ANDSystem and information on the co-occurrence of objects in the literature, representing the results in the form of short digests. However, dictionary-based methods often suffer from low recall, mainly due to their inability to detect novel terms or variations not present in the predefined dictionaries [11,12]. Additionally, these methods may struggle with entity disambiguation, which is required when the same term refers to different concepts depending on the context. The latter problem is especially relevant for the names of genes and proteins [13,14].

Relation extraction aims to identify meaningful relationships between recognized concepts. Traditional relation extraction systems, including ANDSystem [7,8], Pathway Studio [15], and RelEx [16], often employ rule-based approaches in which predefined linguistic patterns and semantic rules are used to extract relationships. The significant advantages of these methods are their interpretability and accuracy, which are crucial in biomedical research where the extracted relationships can directly impact downstream analyses. However, rule-based relation extraction approaches can be labor-intensive and may falter when dealing with complex or nuanced language [6].

Co-occurrence-based methods are another traditional technique widely used to identify relationships, which they achieve by statistically detecting entity pairs that are mentioned within the same context to a significant degree. The main advantages of this class of methods are their ease of implementation and high recall in extracting information. Examples of systems using this approach are ANDDigest [10] and STRING [17]. However, co-occurrence methods have several significant drawbacks, particularly their high false-positive rate. Furthermore, they cannot be applied to entities that are new or poorly represented in the literature [3,9,10].

### 1.2. Deep Learning and Large Language Models in Biomedical Natural Language Processing

Deep learning in natural language processing (NLP) has revolutionized language processing and interpretation. One of the key advantages of deep learning methods is their ability to generalize from vast amounts of data, which is essential for capturing complex linguistic nuances and semantic structures. While supervised learning models benefit from large, well-annotated training datasets, many large language models (LLMs) mitigate this dependency by leveraging self-supervised learning on extensive unannotated text corpora. This approach allows models to learn generalizable patterns from vast amounts of data, capturing the full range of linguistic variations and contextual relationships. However, a significant trade-off exists—deep learning models, especially LLMs, exhibit less interpretability compared to traditional text-mining techniques, making the reasoning behind their decisions often opaque [18].

In the early days, Recurrent Neural Networks (RNNs), particularly Long Short-Term Memory (LSTM) networks, were the go-to architecture for sequence modeling tasks. LSTMs, introduced by Hochreiter and Schmidhuber in 1997 [19], showed a remarkable ability to capture long-term dependencies in sequential data. Deep associative learning approaches based on RNNs were successfully used in applications such as biomedical sentiment analysis utilizing unsupervised representation from large-scale patient narratives [20]. However, even with the advancements of LSTMs, these models faced challenges with very long sequences due to computational inefficiency and difficulties in capturing extremely long-range dependencies. Additionally, their sequential nature hindered parallel processing, leading to slow training times on large datasets.

The landscape changed dramatically with the introduction of the Transformer architecture by Vaswani et al. in 2017 [21]. Transformers brought several advantages: they could process all input tokens in parallel, making them much faster to train. The self-attention mechanism allowed them to capture long-range dependencies more effectively than RNNs. This led to significant improvements in various NLP tasks and paved the way for large language models like BERT and GPT.

Trained on extensive textual datasets, LLMs leverage attention mechanisms to consider the context and positional relationships of individual words (tokens) within a sequence. The attention mechanism enables the model to assign appropriate weights to different words based on their relevance to each other, effectively capturing intricate dependencies and interactions across the entire text. This results in context-dependent vector representations of words, facilitating the identification of complex patterns of relationships between molecular-genetic entities, including entire chains of interactions often overlooked by classical analyses [21,22]. Unlike earlier deep learning NLP techniques, LLMs can analyze entire sequences simultaneously, leading to a more nuanced understanding of language. Leveraging their strong generalization capabilities, LLMs can recognize references to biological concepts even when authors use synonyms that are absent from dictionaries or knowledge bases or that contain misspellings and extrapolate implicitly expressed relationships between them [23,24,25]. Another interesting feature of LLMs is their ability to generate interpretations of obtained results in the form of coherent natural language [26,27].

On the other hand, the evolution of AI methods has led to the emergence of new problems and the actualization of several existing ones related to knowledge extraction. The exponential growth of information presents challenges for data processing and knowledge extraction; studies have highlighted that a significant portion of global data has been generated in recent years, overwhelming traditional processing methods [28]. Another problem introduced by the use of LLMs in NLP is the phenomenon known as hallucination, where the model generates text containing factual claims that are not supported by the training data or literature. This includes connections between molecular-genetic entities, symptoms, and diseases, which have the potential to spread incorrect or misleading information [29,30]. The problem of reliability is particularly acute in medicine, where such errors may have serious consequences for patient outcomes [31].

In the context of NLP tasks, this problem is similar to the incorrect interpretation of connections based on semantic-linguistic rules and patterns, which, in the case of deep learning, are represented by regularities identified by the neural network in large datasets. Unlike manually formed rules, the patterns used by LLMs have weak interpretability. To address interpretability concerns, researchers are developing explainable AI techniques that aim to make the decision-making processes of LLMs more transparent and understandable [18]. One way to mitigate hallucination is to integrate language models with predefined ontological models that contain structured, interpretable knowledge representations, such as graphs [32,33]. This approach allows for filtering objects and relations that go beyond the established ontology, enhancing the accuracy of information extraction.

Another significant challenge of large-scale LLMs, particularly general-purpose models like GPT-4 and Claude, is their high computational resource requirements and generation costs compared to traditional methods [29,34]. This can be mitigated by adapting smaller models tailored to highly specialized tasks or by optimizing input data to reduce computational load while minimizing information loss [35,36].

### 1.3. Graph Neural Networks and Their Application to Biomedical Knowledge Graphs

Graph neural networks (GNNs) have emerged as a promising approach to analyzing structured data in graphs, which are a natural way of representing complex relationships in various domains, including biology and medicine. GNNs extend traditional neural networks by incorporating a graph structure, allowing them to model dependencies and interactions between entities such as proteins, genes, or diseases in a way that corresponds to the inherent topology of the graph [37].

One of the main advantages of GNNs is their ability to learn and propagate information across the nodes and edges of a graph, making them particularly suitable for tasks such as link prediction, node classification, and clustering in biomedical knowledge graphs. These tasks are essential for identifying potential interactions between entities, predicting unknown associations, and uncovering underlying processes in alignment with the graph’s ontology. This interpretability makes GNNs one of the most transparent deep-learning approaches available [38].

In biomedical settings, GNNs have been successfully applied to problems such as predicting protein–protein interactions, drug–target interactions, and disease–gene associations. For instance, Kipf and Welling demonstrated the use of graph convolutional networks for the semi-supervised classification of graph-structured data. This technique has been adapted for predicting protein functions and interactions in biological networks [39].

Advanced models like GraphSAGE [40] and graph attention networks [41] have been introduced to enhance the interpretability and performance of GNNs by allowing them to focus on the most relevant parts of the graph during the learning process. Such approaches have shown promise in applications like drug repositioning and understanding molecular mechanisms of diseases [42].

One of the important tasks in biomedicine is the identification of subgraphs in large-scale biological networks that may represent functional modules associated with specific phenotypic traits, biological processes, or metabolic pathways [43]. This task has been formulated as a combinatorial optimization problem of finding heavy subgraphs, known as the heaviest k-subgraph problem (k-HSP), which is NP-hard. However, methods based on the k-HSP require the parameter k and may yield “spurious” heavy subgraphs, reducing their practicality for large-scale networks. To overcome these limitations, W. Li et al. proposed a new formulation called the rank-HSP, which eliminates the need for the parameter k. They used dynamical systems to approximate the solution of the rank-HSP through two key approaches: Replicator Dynamics (RD) and Bidirectional Associative Memory (BAM) [44]. Important properties of the dynamic behaviors of neural networks and their stability were analyzed in detail by Xu and coauthors [45,46], providing foundational insights that could inform the design of algorithms for network analysis.

The identification of subgraphs in biological networks is closely related to the capabilities of GNNs. While GNNs excel at learning and propagating information across graph structures, they can also be adapted to identify important subgraphs or clusters within larger networks. This capability aligns well with the task of finding functional modules in biological networks, as GNNs can learn to recognize patterns and structures that may correspond to these modules.

However, a significant limitation of GNNs is the need for large and well-annotated graph datasets to train them effectively, which can be challenging to obtain in the biomedical domain. The accuracy of GNN-based models is determined not only by their alignment with the true nature of the interactions but also by their correspondence with the topology of the original graph [47]. With noisy or incomplete data, GNNs may struggle to provide reliable insights beyond the information already captured by the graph [48,49]. Additionally, GNNs can be computationally intensive, particularly for large graphs, which may hinder their scalability and practical implementation in real-world scenarios [40].

### 1.4. Hybrid Approaches

Although individual NLP techniques have limitations regarding their completeness, accuracy, scalability, or interpretability, hybrid approaches can overcome these problems by combining the strengths of multiple methods [50,51]. One example of a hybrid approach is the combination of rule-based methods with machine learning algorithms for enhanced entity recognition and relation extraction. Rule-based components can capture domain-specific knowledge and handle exceptions, while machine learning models can generalize from data and manage variability in language use. For instance, integrating dictionary-based named entity recognition with statistical machine learning methods improves the identification of biomedical entities by recognizing both known terms and novel variations that are not present in dictionaries [52].

Similarly, combining deep-learning models with structured knowledge bases has shown promise in biomedical NLP. Deep-learning models, such as LLMs, excel at capturing contextual information from text. Knowledge bases provide structured information that can guide and constrain the models’ predictions [53,54].

One example of a hybrid approach is work [55]. The approach combined knowledge graph embeddings with a bidirectional long short-term memory (BLSTM) model to uncover hidden associations in biomedical literature, integrating domain-specific knowledge into the discovery process. The knowledge graph provided a structured representation of biomedical entities and their relationships, while the deep learning model captured complex patterns, enhancing the model’s ability to generalize across different contexts. The benefits included improved performance with a better representation of semantic relationships that led to more accurate predictions of drug-target-disease associations, as well as the ability to handle large, complex datasets through dimensionality reduction via graph embeddings.

Another example of state-of-the-art hybrid approaches is a Griffin model that combines gated linear recurrences with local attention, aiming to overcome the limitations of recurrent neural networks (RNNs) and transformer-based models [56]. One of the primary strengths of the Griffin architecture is its ability to handle long sequences more efficiently than traditional Transformers. Griffin achieves enhanced contextual understanding by incorporating local attention without the quadratic computational complexity associated with global attention in transformers. Additionally, the gated linear recurrences allow for capturing long-term dependencies in sequences, which is particularly valuable in natural language processing tasks involving lengthy texts. However, Griffin’s hybrid nature also comes with limitations. While it performs well on most downstream tasks, Griffin exhibits suboptimal performance on specific tasks that require exact copying or retrieval compared to Transformer models. Additionally, combining recurrent layers and local attention can introduce additional complexity in model optimization and may not consistently outperform specialized models designed for particular tasks.

Hybrid methods can also extend to integrating co-occurrence-based techniques with semantic analysis, which can improve the likelihood of finding meaningful relationships between entities while reducing false positives [57]. This provides an enhanced ability to extract relevant information from large corpora of biomedical literature.

In our work with ANDDigest, we have integrated fine-tuned language models with concept recognition methods based on predefined dictionaries. This hybrid approach yields a significant improvement in the accuracy of recognizing short molecular-genetic entity names by an average of 80%, leading to the more precise reconstruction of AGNs. Specifically, the context-based gene and protein name recognition model achieved an ROC AUC accuracy of 0.924 for terms of any length and 0.897 for object names with a length of 4 characters or fewer [10]. Combining the contextual understanding of language models with the specificity of dictionary-based methods, we have addressed entity recognition and relation extraction challenges in complex biological texts.

It should be noted that hybrid approaches come with certain trade-offs. The increased complexity in model design can lead to more challenging development and maintenance processes. Moreover, integrating multiple models often increases computational overhead, making hybrid approaches more resource-intensive in both training and inference compared to single-model systems.

In this study, we present a novel method that combines structured ontology models, graph neural networks, and fine-tuned large language models to efficiently extract knowledge from biomedical literature. Our hybrid approach integrates traditional text-mining techniques (template-based and co-occurrence-based) with advanced deep learning models (GNNs and fine-tuned LLMs), enhancing the prediction of protein-protein interactions and improving result interpretability. This approach extends biomedical knowledge graphs and interprets predicted edges based on the literature, which can significantly accelerate subsequent expert verification. Our method effectively integrates information from both graph and textual representations, providing tools for preliminary validation of predictions and offering explanations for positive predictions made by the GNN based on the context of relevant documents. Our approach also optimizes computational resources, using graph neural networks for initial predictions and language models for targeted validation.

By integrating information about the statistical co-occurrence of human protein pairs in the literature with GNNs, we improved the accuracy of edge predictions relative to the topology of the original graph. Additionally, using a GNN to predict pairs reduces the volume of data passed to the fine-tuned LLM tasked with validating predictions using the document context. This approach provides insights into the existence or absence of relationships based on our ontology, represented by the topology of the associative network and the real biological mechanisms reflected in scientific publications.

Using a dataset containing pairs of human proteins experimentally established as non-interacting, we showed that employing a classification model trained on vector representations of graph vertices in the first step of analysis allows for filtering pairs that deviate from the original ontology. This can reduce the impact of hallucination errors from the LLM and also ensure more efficient use of computational resources when processing large volumes of biomedical data by reducing the number of processed documents, ensuring higher reliability of established edges, and simplifying their validation.

Compared with the previously implemented method in ANDSystem [8], our approach has demonstrated improved effectiveness by identifying interactions between protein pairs related to insomnia that were missed by the original text-mining approach. These advancements have the potential to significantly accelerate therapeutic target discovery and improve our understanding of complex biological systems.

## 2. Results and Discussion

### 2.1. Obtaining Vector Representations of Nodes of the Associative Gene Network for Homo sapiens from ANDSystem Based on a Graph Neural Network

**Figure 1:**
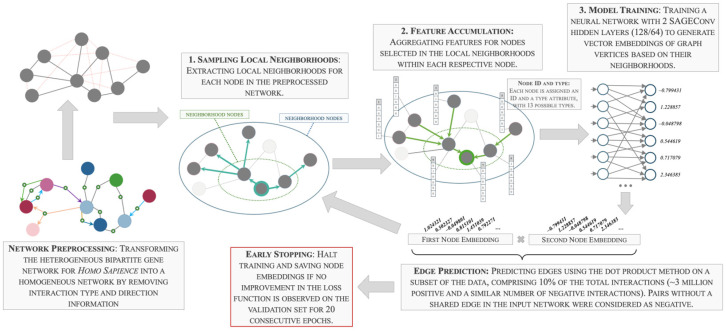
Scheme of GNN training based on the associative gene network (AGN) for *Homo sapiens* from the ANDSystem database.

The GNN was trained using the GraphSAGE method. The general scheme of the process is presented in Figure 1.

The efficiency of this method stems from two main factors. The first is its sampling approach: instead of processing the entire network at once, GraphSAGE samples a subset of each node’s neighbors and part of their local surroundings. This significantly reduces the volume of data processed in each step and thus the computing resources, without a significant loss of accuracy. The second is its inductive learning: because the features of each node are calculated based on its neighborhood, GraphSAGE can generate embeddings for new, previously unseen nodes without needing the entire model to be retrained.

The training process with early stopping criteria ran for 916 epochs. The resulting final model, from which the vector representations of the nodes were obtained, had F1-scores of 0.8016 on the validation set and 0.8034 on the test set.

### 2.2. Training a Binary Classifier Multilayer Perceptron Model Based on Vector Representations of Nodes Obtained via Graph Neural Networks

Given the relatively large size of the training set (200,000 positive and 200,000 negative examples), we trained a classification model based on a multilayer perceptron (MLP) with three hidden layers to predict the probability of a functional association between a pair of proteins. The input data consisted of vector representations of protein pairs obtained from the trained GNN and their corresponding co-occurrence values from the ANDDigest database. The training process, with early stopping criteria based on the Matthews correlation coefficient (MCC) [58] on the validation set, ran for 161 epochs. The final MCC achieved on the validation set was 0.9641; on the test set, it was 0.9535.

To determine the extent to which the co-occurrence values affect the model’s prediction accuracy, we retrained the model from scratch without using co-occurrence values. Slightly lower values of the metric were obtained: the training process lasted for 121 epochs, and on the validation set, the MCC was 0.9529, whereas on the test set, it was 0.939.

### 2.3. Fine-Tuning the Model to Predict and Explain Functional Interactions Between Protein Pairs Based on the Context of Documents Mentioning These Pairs Together

**Figure 2:**
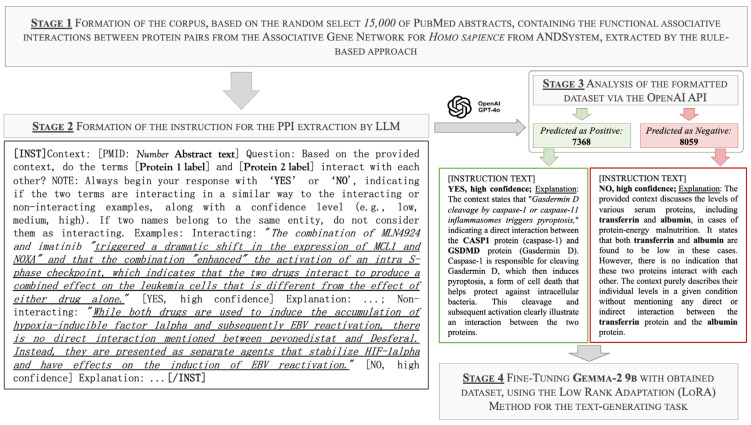
Scheme used to fine-tune the Gemma-2-9b-it LLM for the task of predicting functional associations between protein pairs based on the context of documents mentioning them.

To verify the predicted interactions and interpret them based on document context, we performed the fine-tuning of the pre-trained LLM Gemma-2-9b-it [59] for the language modeling (LM) task (Figure 2).

The choice to use pre-trained language models that perform text generation rather than classical binary sequence classification was made due to the possibility of obtaining not only information indicating the presence or absence of interaction between a pair of proteins. It also allows for a concise explanation of the conclusion made, including, in the case of a positive answer, the biological mechanism of the interaction.

The training lasted for 100,000 iterations, corresponding to approximately ten epochs, with intermediate results saved every 10,000 steps and a learning rate of 1.000 × 10^−5^ (the default value). The proportion of trainable parameters for the trained low-rank adaptation (LoRA) adapter was 0.047% of the total model parameters (4.473 million out of 9241.706 million). The metric used for accuracy assessment was the loss function; the best loss values were achieved after 70,000 steps, amounting to 0.709 for the validation set and 0.688 for the test set, with 62,216,326 trained tokens over all epochs.

It should be noted that the obtained loss metric values are not highly informative because, when fine-tuning for the language-modeling task, the loss function measures the model’s ability to predict words (tokens) in the sequence, including the text of the documents themselves. This may not directly reflect the model’s performance in predicting protein interactions. Therefore, we used two well-known gold standards to assess the accuracy of the model’s predictions of interactions between protein pairs: IEPA [60] and HPRD50 [16]. The detailed process of forming the samples and the rationale for the choice of Gemma-2 as the initial LLM for fine-tuning are described in Section 3.2.3 and Section 3.2.4.

### 2.4. Evaluation of Protein–Protein Interaction Prediction Accuracy Based on Gold Standards Using a Fine-Tuned Model

In the output format we used for the fine-tuning and evaluating of the model, any generated answer should always begin with “YES” if the model identifies the presence of an interaction between a pair of proteins in the document context, or “NO” otherwise. Thus, all assessments of correct and incorrect predictions were based on the presence of one of these word indicators located at the beginning of each generated sequence.

Applying our fine-tuned model to the formed samples based on the IEPA and HPRD50 gold standards (the total input and output data of the model for each standard are provided in Supplementary Files S1 and S2) resulted in approximately equal metric values. Specifically, for IEPA, the MCC was 0.844, whereas for HPRD50, it was 0.8422; more detailed information is provided in Table 1. In both cases, for negative examples, we used randomly selected unique pairs of human proteins from the Stelzl2005 list [61] of pairs that showed no interactions during experimental verifications. This was compiled by its authors using information from the IntAct molecular interaction database [62].

A manual analysis of the model-generated responses that were labeled as incorrect showed that most of the documents for which relationships between entity pairs were misrecognized dealt with indirect interactions. In these cases, one protein influences the activity or production of another protein through intermediaries or signaling pathways. For example, in the study by Molnár and Hertelendy [63], which was presented in the IEPA gold standard as containing an interaction between oxytocin and IP3 proteins, our fine-tuned model concluded that there is no interaction between them. However, the authors stated that oxytocin activates the phospholipase C–IP3–Ca2+ pathway in human myometrial cells. Although it does not directly bind to IP3, oxytocin initiates a signaling cascade that results in IP3 production, implying an indirect interaction.

The model generates a similar conclusion regarding the absence of interaction between the same pair of proteins when analyzing the text of the study by Marc et al. [64]. Even though the title of this work states that “oxytocin stimulates the generation of inositol phosphates in the guinea pig myometrium”, the text of the document describes the interaction between oxytocin and IP3 proteins as indirect: oxytocin activation leads to IP3 generation through the activation of phospholipase C.

Similarly, in [65], the authors stated that buspirone enhances the oxytocin response during insulin-induced hypoglycemia. The model concluded that there was an absence of direct interaction between oxytocin and insulin proteins. However, while they do not directly interact with each other, the study found that insulin’s effect on oxytocin levels signifies an interaction in the context of hormonal regulation.

Most of the false positive interactions were related to a similar situation. The texts that led to the most such errors involved complex regulatory pathways with multiple proteins influencing a common process or complex, such as situations in which several proteins form a complex (e.g., the Mis12 complex [66], the APC/C complex [67]), but not all components interact directly. However, the high values of the performance metrics indicated the ability to use this model within our software pipeline.

### 2.5. Assessing the Hybrid Approach: Protein-Protein Interaction Prediction Performance Compared to Leading Large Language Models

To evaluate our developed approach in comparison with existing state-of-the-art methods and assess the impact of our trained graph neural network on the final results, we conducted a comparative analysis. In addition to our fine-tuned language model (Gemma-2-9b-it-Fused_PPI), we selected five pre-trained LLMs: o1-preview [68] (OpenAI, San Francisco, CA, USA), Claude-3.5 Sonnet [69] (Anthropic PBC, San Francisco, CA, USA), Command-R-Plus (command-r-plus-08-2024) [70] (Cohere Inc., Toronto, ON, Canada), Gemini-1.5-Pro [71] (Google LLC, Mountain View, CA, USA), and Mistral Large (mistral-large-2407) [72] (Mistral AI, Paris, France). These models are considered leaders among LLMs in solving tasks requiring complex analysis and data interpretation, including biology and biomedicine [73,74,75].

As in the previous evaluation, we used the HPRD50 gold standard dataset, which had the best overlap with our ANDSystem ontology: 129 protein-protein interactions out of 152 in the positive sample. The negative sample remained the same as in Section 2.4. The evaluation was conducted in two ways: first, we directly compared the results of our fine-tuned model with the listed LLMs. Second, we introduced a preliminary classification step, where we used our trained graph neural network to classify protein pairs based on their vector representations before passing them to the language model prediction stage.

**Figure 3:**
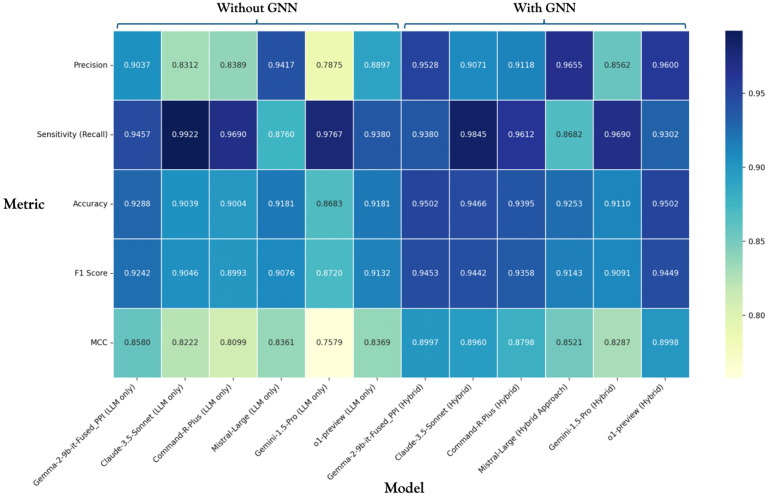
Evaluation results of the PPI identification task on the HPRD50 dataset by selected LLMs with and without preliminary classification of interactions using a graph neural network.

The results of the comparison are presented in Figure 3. The heatmap shows each model’s performance metrics (Accuracy, F1-score, MCC, Precision, and Sensitivity), both with and without the preliminary classification step using the developed GNN.

The results demonstrate that in all cases, adding the interaction filtering step based on the ontological model topology improved the Accuracy, F1-score, and MCC values. This improvement was achieved by reducing the number of false positive examples passed to the language model, as evidenced by the increased Precision. Notably, the decrease in Sensitivity (true positive rate) was minimal across all models.

Our model, which was fine-tuned specifically for this task, showed one of the best performances both with and without the graph neural network preprocessing step, being slightly inferior only to the latest o1-preview model from OpenAI (San Francisco, CA, USA) when used together with the GNN in the hybrid approach.

It is worth noting that the improvement in performance was most pronounced for models that initially had lower Precision. For instance, the Claude-3.5 Sonnet, Command-R-Plus, and Gemini-1.5-Pro models, which had the lowest initial Precision values, showed significant improvements when combined with the graph neural network approach.

These findings suggest that our hybrid approach is particularly effective when applied to language models that tend to over-predict interactions. By incorporating the topological information from the knowledge graph, we can significantly reduce false positives while maintaining high Sensitivity, resulting in a more balanced and accurate prediction of protein-protein interactions.

### 2.6. Evaluation of the Accuracy of the Developed Software Pipeline in Predicting Protein–Protein Interactions Based on Experimental Data

To assess the accuracy of the entire method on experimental data, we compiled a corpus of 4190 examples of human protein–protein interactions, divided into experimentally confirmed positive and non-interacting pairs in a 1:1 ratio. As in the previous case, information about non-interacting pairs was obtained from the Stelzl2005 list, whereas positive interactions were extracted from the IntAct database. The detailed process of forming the corpus is described in Section 3.2.5. Processing the data using our MLP binary classifier indicated that 1704 of the 2095 positive pairs (81.3%) were interacting. A pair was considered interacting if the predicted value exceeded a threshold of 0.5. For non-interacting pairs, the MLP model indicated interactions for only 47 (0.02%).

Next, a corpus of PubMed abstracts was compiled using the ANDDigest database for all the protein pairs (positive and negative) that were predicted as interacting. The 1704 positive interactions were mentioned in 49,102 documents 60,084 times. Application of the fine-tuned LLM revealed that 1571 of the 1704 analyzed pairs (92.19%) had documents with context indicating interaction. For negative examples, among the 47 pairs classified as interacting, 40 pairs were mentioned 53 times in the 53 abstracts. Analysis of the context using the LLM showed that only three pairs (7.5%) were defined as functionally related.

However, one error appeared to originate not from the model’s performance but from inaccuracies in the annotation of names within ANDDigest itself. The phosphoprotein phosphatase 1 catalytic subunit gamma 2 (PPP1CC2) is a protein essential for spermatogenesis and sperm motility [76]. It was recognized as tryptophanyl tRNA synthetase due to the absence of the PPP1CC2 isoform of PPP1CC in the ANDSystem’s dictionary and the presence of gamma 2 among the synonyms of tryptophanyl tRNA synthetase. In two other cases, the decision about the interaction was made based on the involvement of the pairs of proteins in shared pathways.

Thus, our method allowed the reconstruction of 75% of the initial number of true positive interactions, with an overprediction error of less than 1% (for detailed information see Supplementary Files S3–S5); the MCC value was 0.772. The achieved metrics are similar to those of the original ANDSystem graph, which had a high precision value with a recall of less than 60% [7].

### 2.7. Application to Insomnia as an Important Urban Health Challenge

Insomnia is a widespread health problem, particularly in urban environments, where a confluence of negative factors such as environmental stressors (e.g., excessive exposure to artificial lighting, air, and noise pollution) [77], unhealthy lifestyles (e.g., low physical activity, unbalanced diet), and socioeconomic pressures [78]. Common aspects of urban lifestyles, such as high levels of stress, irregular work schedules, and excessive exposure to artificial lighting, can negatively affect the natural human circadian rhythm, contributing to the development of chronic insomnia [79]. Another factor is the influence of digital device screens, promoting overexposure to blue light and encouraging late-night engagement in stimulating activities, which are detrimental to sleep onset and maintenance [80].

Understanding the molecular mechanisms underlying insomnia is essential for developing preventive strategies. From the perspective of systems biology, insomnia can be defined as a complex disorder arising from the dysregulation of interconnected biological networks involving circadian rhythms, stress responses, and neuroplasticity. These factors involve interactions between numerous proteins, so it is necessary to reconstruct comprehensive associative networks to elucidate the pathophysiology of insomnia. Among the most important components of these networks are protein–protein interactions, which play a fundamental role in cellular function and signaling pathways.

Given the complexity and multifactorial nature of insomnia, a comprehensive approach based on the analysis of large volumes of biomedical data are needed for a deeper understanding of its molecular mechanisms. As part of our research, we applied our software pipeline to identify new relationships between proteins potentially involved in the pathophysiology of insomnia, which, combined with the information presented in the ANDSystem knowledge base, can serve as a basis for developing new therapeutic strategies.

**Figure 4:**
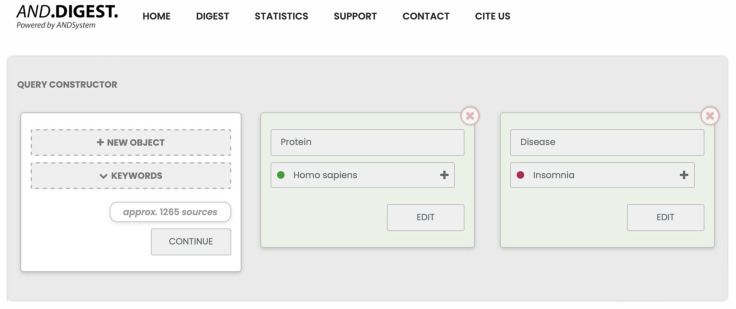
Illustration of the ANDDigest query based on the ANDSystem ontology for searching documents that simultaneously contain the term “Insomnia” and at least one human protein.

In the first stage, through the ANDDigest web interface, we formulated a query to search for PubMed documents that mentioned insomnia and at least one human protein (Figure 4). A total of 1240 abstracts were found, mentioning 625 proteins. During result generation, the ANDDigest system also automatically marks up any other molecular genetic entities encountered in sentences that satisfy the original query and are consistent with the ontology. Because of this, some proteins from this list related to other organisms were filtered out.

**Figure 5:**
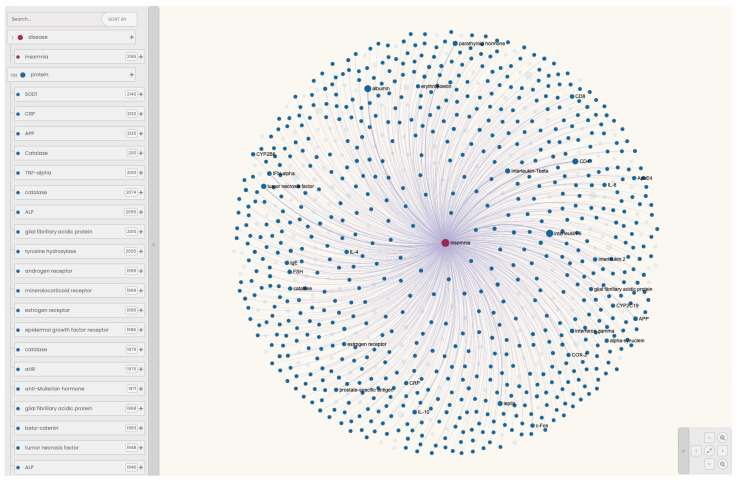
Network of human proteins with statistically significant associations with insomnia in the literature, reconstructed using the ANDDigest web interface. The size of each sphere corresponds to the frequency of occurrence of the object in the obtained corpus of documents. Transparent spheres indicate proteins filtered based on *p*-values.

Using the ANDDigest web interface, we also filtered out those human proteins whose co-occurrence with insomnia was not statistically significant (co-occurrence *p*-value > 0.05). Thus, the final list contained 131 objects (Figure 5), with complete information presented in Supplementary File S6.

**Figure 6:**
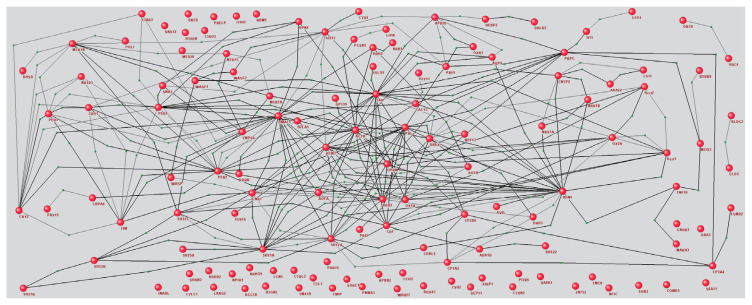
AGN of interactions between proteins with statistically significant co-occurrence with insomnia in PubMed abstract texts, reconstructed in ANDSystem.

Since we were interested in the applicability of our method to predicting interactions overlooked in the ANDSystem network, to exclude previously identified ones, we reconstructed an AGN (Figure 6). The resulting network contained 242 edges between 90 of the selected 131 proteins that co-occur with insomnia in the literature to a statistically significant degree.

**Figure 7:**
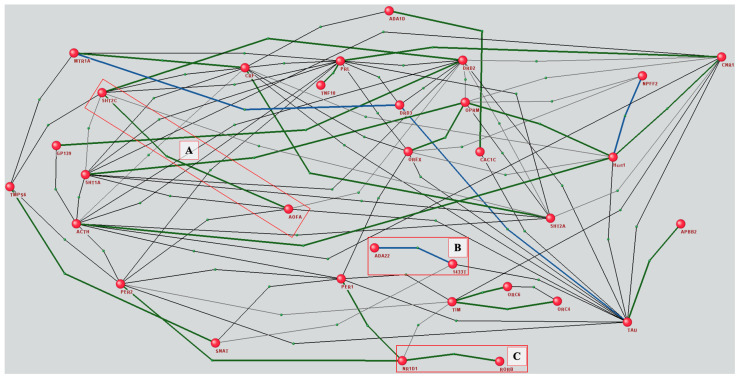
Subgraph of the reconstructed AGN based on protein pairs with statistically significant associations with insomnia expanded with new edges. Thin black lines correspond to connections from the original network, thick lines indicate new interactions discovered using the software pipeline, green lines correspond to regulatory relationships, and blue lines indicate physical binding.

In the next step of our analysis, based on this list of proteins, the AGN was reconstructed, describing known interactions between them (Figure 6). We aimed to predict missing connections and thus expand the AGN. Based on the reconstructed AGN, a set of all pairs of unconnected network vertices was compiled, totaling 8388 pairs. The graph-based classification model showed that 6240 pairs could potentially be interacting. Among these, 657 were represented in ANDDigest, mentioned 4350 times in total by published works. A subsequent analysis with an LLM confirmed 25 interactions between 32 human proteins that were absent in ANDSystem (Figure 7).

Verification of the sources in which functional associations confirming predicted interactions were discovered showed that twenty of them were regulatory in nature and three protein pairs were physically linked. For the remaining two pairs, the connection was established based on their co-localization. The full list of new interactions, including the interpretations generated by the language model based on context and manual classification, is presented in Supplementary File S7.

In particular, from the work of Ni et al. [81], which focused on studying a number of serotonin genes and their interactions in patients suffering from borderline personality disorder, the software pipeline identified a significant interaction between monoamine oxidase A (MAOA) and the serotonin receptor 2C (5-HT2C; box A in Figure 7). The MAOA gene controls the availability of serotonin by breaking it down, whereas 5-HT2C is a G protein-coupled receptor that binds serotonin and mediates various neurological processes. Alterations in MAOA activity can lead to changes in serotonin levels, which directly affect the activation of 5-HT2C receptors. Serotonin is an important and well-studied neurotransmitter involved in regulating sleep–wake cycles and sleep architecture [82]. Disruptions in the regulation of this neurotransmitter, as well as receptor functions, can lead to various disturbances in human behavior, including insomnia and depression [83,84].

Another example is the connection between the enzyme ADAM22 and 14-3-3 proteins (box B in Figure 7). In [85,86], the authors demonstrated for the first time an association between ADAM 22 and a 14-3-3 protein. This interaction is of interest because ADAM 22 has been implicated in various neurological diseases, such as epilepsy, encephalopathy, and neurodegenerative conditions, among the side effects of which insomnia is present [87,88]. In turn, the 14-3-3 protein family, particularly 14-3-3ε, plays a significant role in regulating circadian rhythms and sleep patterns [89,90,91]. Further research into their interaction, particularly in sleep-related brain regions, could provide new insights into the mechanisms of sleep–wake cycles and stress-induced insomnia.

Based on the work by Hou et al. [92] on the molecular mechanisms of sleep duration in the Taiwanese population, a regulatory connection between the circadian clock genes RORB and NR1D1, previously absent in ANDSystem, was identified (box C in Figure 7). The work presented data showing that the mutual regulation between RORB and NR1D1 genes forms a secondary feedback loop in the circadian rhythm machinery, with RORB activating NR1D1 expression while NR1D1 represses BMAL1 and RORB expression. This identified interaction may play a significant role in the stability and precision of the circadian clock, directly influencing sleep duration.

## 3. Materials and Methods

### 3.1. The Overall Scheme of Our Hybrid Approach

**Figure 8:**
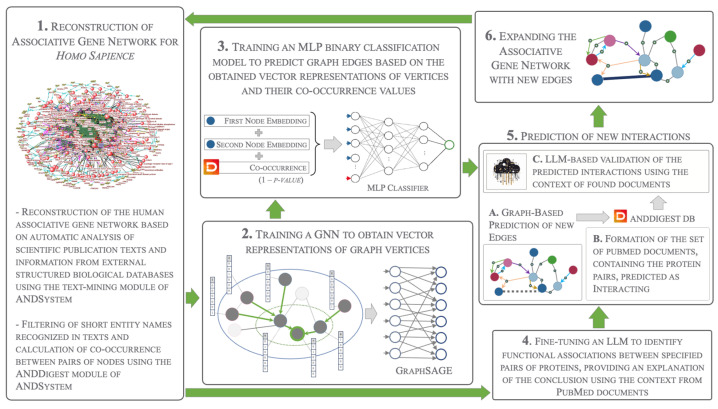
General scheme of the proposed method for predicting interactions between pairs of proteins, based on the combined use of graph neural networks (GNNs), statistically significant co-occurrences of pairs in the literature, and large language models (LLMs).

The overall scheme of the developed software pipeline is presented in Figure 8. In the first step, we performed an initial reconstruction of the knowledge graph for the human organism, following the protocol for automatic knowledge extraction previously implemented in ANDSystem [7]. This included filtering out short names (with four characters or less) and calculating pairwise co-occurrence values for all entities from the ANDSystem ontology, annotated in the corpus of PubMed abstracts using the ANDDigest module [10].

Vector representations for each node of the reconstructed knowledge graph were then obtained by training a GNN using the GraphSAGE approach [40]. Next, a binary classification model was trained to predict the presence of interactions between pairs of human proteins on the basis of their co-occurrence values and the obtained vector representations. We fine-tuned an LLM, Gemma-2-9b-it [59], to generate text that predicts interactions between pairs of objects identified on the basis of the semantic-linguistic rules and patterns of ANDSystem. We used the context of documents in which these interactions were mentioned to explain their mechanisms.

Based on the graph’s topology, the classification model was applied to predict the probability of interactions between pairs with missing edges in the associative network. An automatic search in ANDDigest was conducted for literature sources mentioning pairs of objects predicted to interact, and the fine-tuned model was applied to these pairs to verify the presence of a connection based on the context of these documents.

Finally, the method was applied to pairs of proteins related to insomnia, expanding the original network with new edges that were missed by our original text-mining approach implemented in ANDSystem. Each new edge was accompanied by a description of the interaction mechanism. This facilitates subsequent manual verification of the predicted interactions.

### 3.2. Data Preparation

#### 3.2.1. Formation of the Initial Graph

The initial graph, describing molecular-genetic interactions at the whole-genome level of humans, was extracted from the ANDSystem knowledge base [9,10]. ANDSystem has been successfully applied to the reconstruction of AGNs and the interpretation of genomic, proteomic, and metabolomic data in various fields of biomedicine [93,94,95,96,97,98,99,100,101] and agrobiology [102,103,104]. The resulting bipartite heterogeneous graph included 193,015 vertices of 11 types, connected by 30,702,487 edges corresponding to 36 types of interactions. Detailed statistics on the types of objects and connections are provided in Appendix A Table A1 and Table A2. This network was converted into a unipartite monogenic format and stored in two CSV files containing information about the edges and nodes of the graph, respectively.

#### 3.2.2. Formation of a Dataset for Training a Binary Classification Model to Predict Interactions Based on Vector Representations of Protein Pairs

For the positive examples, we used 200,000 randomly selected protein pairs from the ANDSystem graph, pre-processed as described in Section 3.2.1. These were matched with 200,000 negative examples from the Stelzl2005 dataset [61], which contains 894,213 negative (non-interacting) protein–protein interactions for Homo sapiens derived from large-scale two-hybrid experiment data from the IntAct database. The negative examples were also randomly selected. Each member of a pair was represented by its vector representation, based on the topology of the original ANDSystem graph, with a length of 32, along with the value of their co-occurrence. Co-occurrence values for the positive examples were obtained from the ANDDigest module database, based on the analysis of PubMed abstract annotations using dictionaries, whereas those for the negative examples were randomly selected from all human protein pairs in the ANDDigest database. These values were calculated using the *p*-values with the formula 1 − *p*.

#### 3.2.3. Selection of a Pre-Trained Model and Formation of a Training Sample for Fine-Tuning the Prediction of Protein Pair Interactions Based on the Document Contexts

The choice of the Gemma-2-9b-it model, among the wide range of available LLMs, was dictated by several factors. The Gemma-2 models [59] (Google LLC, Mountain View, CA, USA) incorporate the advanced grouped query attention mechanism [105], which efficiently reduces the computational overhead associated with traditional multi-head attention mechanisms. This allows the models of this class to process queries more rapidly during inference while preserving performance quality, making them particularly suitable for real-time applications in which both computing speed and accuracy are important. Additionally, the model leverages an interleaved attention architecture that alternates between local sliding window attention and global attention layers. This dual-focus approach enables the model to capture both fine-grained local dependencies and broader global contextual information within input sequences, significantly enhancing its ability to understand and generate complex language patterns. The use of logit soft-capping further stabilizes the training process by preventing extreme logit values, reducing potential disruptions during model training, and leading to better generalization.

Another key advantage of the Gemma-2 models is their training regimen, which includes knowledge distillation from larger models [106]. This method replaces the traditional next-token prediction with a richer training objective, allowing the smaller 9B model to achieve performance levels that are competitive with those of significantly larger models such as GPT-4 (OpenAI, San Francisco, CA, USA) and Claude 3.5 (Anthropic PBC, San Francisco, CA, USA). This makes Gemma-2-9B both computationally efficient and exceptionally effective across a broad range of benchmarks.

To prepare training data from the ANDDigest database, we randomly selected over 15,000 PubMed abstracts with dictionary-mapped protein names, containing unique 6516 protein pairs with an edge in the associative ANDSystem network described in Section 3.2.1. The obtained corpus was split into positive and negative parts; we considered documents in which an interaction was identified by the rules-based approach of ANDSystem’s text-mining module as positive, whereas documents in which a pair of proteins were simply mentioned together were considered negative.

**Figure 9:**
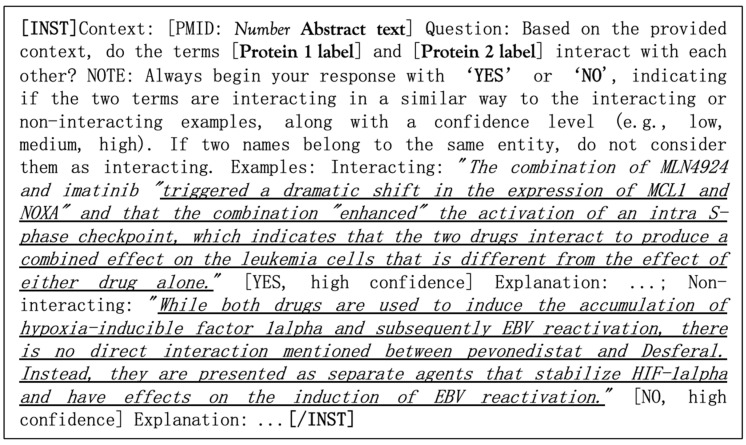
Example of the instruction format for the LLM applied to scientific publication abstracts.

LLMs are typically capable of generating more accurate answers and adhering more closely to output formats when the user query is accompanied by at least a few examples [107,108,109]. Taking this into account, the corpus of texts was reformatted into instructions including the following components: the document context from ANDDigest; a question about the interaction between the protein pair with instructions to use only the provided context for the answer; a confidence level (high, medium, low) for the generated answers; and an explanation for the conclusion. This allows increasing the priority of input tokens over connections stored in the model’s “memory”. To enhance accuracy, we included two examples of correct answers using drug–drug interactions to avoid bias toward specific protein interactions. An example of the instruction format is presented in Figure 9.

The reformatted corpus of documents was passed as input data to the pre-trained LLM gpt-4o-2024-05-13 (GPT-4o), which has a context window size of 128,000 tokens and was, at the time of this study, one of the most advanced models of the fourth-generation GPT family. This was done through the ChatCompletion function, which allows the generation of natural language responses based on a sequence of input messages from the official application programming interface (API) of the OpenAI library version 1.34.0 (OpenAI, San Francisco, CA, USA) for the Python programming language. In one iteration, exactly one example was passed to the model input. The maximum size allowed for the generated output tokens was 256. The value of the temperature hyperparameter, which regulates the level of stochasticity when sampling the next token from the probability distribution obtained at the neural network output, was 0.3. This modification was made to strengthen the model’s tendency to follow the instructions given in the prompt when generating token sequences. The other parameters were set to the default values.

The choice of GPT-4o was due to the high accuracy of its predecessor (GPT-4-1106-preview) compared with other LLMs when working with biological texts, including PubMed [110]. However, despite its high efficiency, the use of commercial models like GPT-4o in tasks of mass literature analysis in scientific research is limited by the high cost of token generation as well as the limits on the total number of tokens set by companies [111]. Another reason for using generated data were that such synthetic data usually have a clearer overall structure than human-written text. Therefore, when training smaller models on such data, it is easier for them to detect signals and establish patterns. This approach, known as knowledge distillation [106], involves using results generated by larger models as target values for further training and fine-tuning of smaller models. This opens up opportunities for more effective application of the latter in solving a wide range of specialized tasks, such as information retrieval and summarization, compared with large models. In particular, the effectiveness of this approach was demonstrated in [112], which used the Alpaca model as an example and obtained better results after knowledge distillation from larger language models.

After the manual selective result verification, these token sequences generated by GPT-4o were used as ground truth examples in training samples. The obtained dataset contained 7368 positive examples and 8058 negative examples.

#### 3.2.4. Formation of Samples for Evaluating the Accuracy of Predicting Protein–Protein Interactions Based on Document Text Using a Fine-Tuned Large Language Model

To evaluate the model’s accuracy, two datasets were formed based on known corpora. Each formed corpus contained an equal number of positive and negative examples. The structure of each example consisted of a list of protein name pairs, their identifiers, and the text of a document from the PubMed database mentioning the corresponding pair, in the instruction format presented in Figure 9.

Positive examples included texts describing the interaction between the corresponding proteins. As negative examples, randomly selected unique pairs of human proteins from the Stelzl2005 list [61] were used, which were non-interacting during experimental verifications. Each such pair was linked to the text of an abstract that mentioned it, extracted from the ANDDigest database. The choice of text among all documents in which the considered pair was present was made at random.

Formation of the Corpus-Based on HPRD50

The first corpus of positive examples of protein–protein interactions was based on the HPRD50 gold standard developed by Fundel et al. [16]. This is a collection of randomly selected and manually annotated abstracts of biomedical publications describing various types of interactions between protein pairs. Data were extracted from the BigBio repository [113] using the load_dataset(“bigbio/hprd50”, “hprd50_source”) function of the Datasets library (version 2.21.0) for Python. The preprocessing included the following steps:

- Removal of documents containing only entity name markup without information about the presence of interaction;
- Loading of document texts from PubMed based on the identifiers presented in the gold standard using the Entrez function from the Biopython package (version 1.84);
- Extraction of lists of mentioned protein names and their identifiers from the entities field of the gold standard;
- Establishment of interactions based on information in the relations field for pairs with interaction type “PPI”;
- Conversion of extracted information into the format of instructions used as input data for the fine-tuned LLM, according to Figure 8.
- The final set of positive examples from the first corpus included 34 unique documents containing 152 protein pairs annotated as interacting.

Formation of the Corpus-Based on IEPA

The source of positive interactions for the second corpus was the Interaction Extraction Performance Assessment (IEPA) gold standard [60]. Information was extracted from BigBio using load_dataset(“bigbio/iepa”, “iepa_source”) followed by similar preprocessing as used for the first corpus. The resulting set of positive examples included 119 unique documents and a total of 295 pairwise interactions between proteins.

#### 3.2.5. Formation of Samples for Evaluating the Accuracy of the Entire Software Pipeline Using the Experimental Data

The positive set of protein–protein interactions was extracted using the IntAct DB web interface (accessed on 11 July 2024) with the following query: https://www.ebi.ac.uk/intact/search?query=species:9606&interactorTypesFilter=protein&interactionTypesFilter=physical%20association,direct%20interaction&interactionHostOrganismsFilter=Homo%20sapiens. Direct and associative interactions between human proteins were considered. The resulting list of interactions contained 4481 pairs. However, 2386 examples that were included in the training sets of the LLM and the MLP classifier were excluded. Thus, the final positive list consisted of 2095 protein pairs whose interactions were confirmed during experimental verifications.

The list of negative examples, representing proteins between which interactions were not detected during large-scale two-hybrid experiments, was formed by randomly selecting the same number of pairs from the Stelzl2005 dataset. As in the previous case, the selection condition was the absence of an intersection with the training samples.

### 3.3. Model Training

#### 3.3.1. Training the Graph Neural Network

The GNN was trained using a Python script implementing the GraphSAGE [40] model for predicting interactions between pairs of vertices in a graph using the PyTorch Geometric (PyG) library (version 2.5.3) [114]. Information about the edges and vertices of the ANDSystem graph was loaded from tables stored in separate CSV files. Normalization of graph vertex features was performed using the StandardScaler function from the Scikit-learn library (version 1.5.0). To split the data into training, validation, and test sets, the RandomLinkSplit function from torch_geometric.transforms was used with the following hyperparameter values:

- is_undirected = True, so that all edges were considered undirected;
- num_val = 0.1, meaning that 10% of edges from the original graph were used for validation;
- num_test = 0.05, so the test set comprised 5% of all edges;
- add_negative_train_samples = True, meaning that the generation of negative interaction examples was carried out using pairs of vertices between which edges were absent in the original graph.

Our GraphSAGE-based model has a two-layer architecture, incorporating node feature aggregation through graph convolutions and link prediction by calculating similarity scores between node embeddings using the dot product method. The model consisted of an input layer and two hidden SAGEConv layers, used to process the graph data progressively. The first convolution layer transformed the input features into 128-dimensional hidden representations, and the second layer refined these hidden representations into 64-dimensional final embeddings.

The “encode” function of the model applied these convolutions sequentially. It first passed the input features and edge indices through the initial SAGEConv layer, followed by a rectified linear unit (ReLU) activation function for non-linearity. The resulting activations were then processed by the second SAGEConv layer to generate the final node embeddings. The training process was performed using the Adam optimizer with weight decay (AdamW), which improves the generalization capabilities of LLMs [115], with a learning rate set to 0.01. The loss function was calculated using the binary cross-entropy with the logits method (BCEWithLogitsLoss).

During the training process of the GNN, link prediction was conducted by assessing the probability of an edge existing between two nodes. This was accomplished by computing the dot product [116] of the embeddings corresponding to each node, by the formula

$$s(u,v)=\sum_{i=0}^{n}\left(z_{u}\left[i\right]*z_{v}\left[i\right]\right)$$

where the variables are defined as follows:

- $s\left(u,v\right)$ is the score for the edge between nodes u and v;
- $z_{u}$ is the embedding vector for node u;
- $z_{v}$ is the embedding vector for node v;
- n is the dimension of the embedding vector.

The early stopping mechanism had a patience of 20 epochs and was based on the improvement of the validation loss value. This early stopping approach allowed the training process to continue if the model was showing improvement on the validation set but stopped it when the model’s performance plateaued or began to degrade, which we considered an indication of overfitting. The model’s performance was evaluated using F1-score metrics on validation and test sets.

#### 3.3.2. Training the Binary Classification Model Based on a Multilayer Perceptron

The model was trained with a Python script implementing a standard MLP architecture for binary classification using the PyTorch library (version 1.13.0) [117]. The model architecture consisted of an input layer, three hidden layers with 256, 128, and 64 neurons, respectively, followed by the ReLU activation function, and an output layer, represented by a single neuron indicating the presence or absence of a connection. The total size of the input layer was 129 neurons, with 128 allocated for the obtained vector representations of the first and second graph vertices, and the value of the last neuron was calculated using the formula 1 − *p*, based on a *p*-value corresponding to the co-occurrence of the relevant pair in PubMed abstract texts, which was taken from the ANDDigest database.

The conversion of model outputs to predictions indicating the presence or absence of an edge for a pair of vertices was performed using the sigmoid function. Prediction values above 0.5 were considered positive. The data were loaded from the CSV files and split into training, validation, and test sets. As with the previous model, the MLP was trained using binary cross-entropy loss and the AdamW optimizer with a learning rate of 0.001. The training process incorporated batch processing with a batch size of 64 and the early stopping mechanism. The early stopping criteria were based on the Matthews correlation coefficient (MCC) on the validation set, with a patience of 50 epochs. The best model was evaluated on the test set.

#### 3.3.3. Fine-Tuning of the Large Language Model for Text Generation

The pre-trained model was fine-tuned using the mlx.lora program from the MLX-LM package (version 0.16.1) [118], which is designed for running and fine-tuning LLMs locally on Apple Silicon processors (Apple Inc., Cupertino, CA, USA). The LoRA method was used as the fine-tuning technique. This method allows efficient adaptation of a model to a specific task while significantly reducing the number of trainable parameters. It injects trainable rank decomposition matrices into each layer of the transformer architecture, keeping the pre-trained model weights frozen, thereby reducing the memory and computational costs associated with the classical fine-tuning of large models. This approach ensured that the fine-tuned model remained highly efficient, both in terms of performance and resource usage, while maintaining the quality of the predictions [119].

The parameters were set as follows: all layers of the model were adapted, the number of training iterations was 50,000, and the learning rate for the optimizer was 2.5 × 10^−5^. The values of the LoRA configuration file are provided below:

- rank = 8; this is the dimensionality of the low-rank matrices added to the model’s layers during adaptation;
- alpha = 16; this is a scaling factor applied to the low-rank updates to determine their influence on the final model;
- dropout = 0.0, meaning that no units were dropped during training, allowing all neurons to contribute fully;
- scale = 10.0, meaning that the magnitude of the low-rank updates was scaled by a factor of 10 to maintain a balance between the pre-trained weights and new task-specific adaptations.

## 4. Conclusions

With the rapid growth of biomedical literature and the advancement of LLMs, integrative and interpretable approaches are playing an increasingly important role in knowledge extraction and deepening our understanding of complex biological systems. The development of deep machine learning methods, such as GNNs and LLMs, has significantly expanded the possibilities for extracting knowledge from biomedical literature, allowing for the analysis of broader contexts and the identification of complex interaction patterns often overlooked by classical text analysis approaches. However, this progress has also brought new challenges, such as increased volumes of processed information, risks of hallucinations when LLMs are used to perform contextual analysis, and non-linearly growing requirements for computational resources, with increased time spent on data processing. Furthermore, the accuracy of GNN-based models depends heavily on the quality and completeness of the underlying graph data. In cases where data are noisy or incomplete, GNNs may struggle to provide reliable insights, which underscores the need for well-annotated and comprehensive datasets. Moreover, although LLMs may enhance interpretability, they still operate as “black boxes” to some extent, making it challenging to validate their predictions [120].

The integration of LLMs with AGNs appears to be one of the most promising directions in the development of NLP methods in bioinformatics, opening a variety of new opportunities for the analysis and interpretation of large volumes of biological data. In this study, we propose a new approach that combines the strengths of LLMs with structured ontological models, statistical co-occurrence of objects, and GNNs. By combining advanced NLP techniques with graph-based models, we have created a method that predicts interactions with high accuracy while providing meaningful explanations for these predictions within the framework of domain ontology and the context of the documents. This allows for reducing the volume of analyzed information, decreasing the computational time required for its processing, which is especially important when using LLMs.

The modular nature of the developed pipeline allows for its parts to be used both sequentially and independently. In particular, the fine-tuned language model can be applied to the original graph for secondary confirmation of interactions, primarily of a functional-associative nature, established through semantic templates. In turn, the GNN can be used both to predict new connections and to weigh existing edges.

However, our approach has limitations related to the use of predefined ontologies that contain a limited number of molecular-genetic objects. Reliance on a fixed ontology means that our model may not account for new or less-studied biological entities that are not yet included in the existing ontological framework. This constraint can lead to incomplete analysis and potentially overlook novel interactions or discoveries emerging from recent research. As a result, the applicability of our method may be restricted when dealing with cutting-edge data that introduces new molecular-genetic objects, or synonyms, not represented in the predefined ontology.

An important direction for further development of our approach is extending it to specifying the type of interactions, including their directions or functional outcomes, with subsequent integration into ANDSystem’s text analysis protocols and its scientific literature search module, ANDDigest. This includes applying this approach to other types of biomedical entities besides proteins, such as drugs, diseases, metabolites, and pathways.

The application of our hybrid GNN-LLM method in biological computer tools, such as the ANDSystem—a system widely used for practical tasks in biomedical research—will significantly enhance capabilities in drug discovery, therapeutic target identification, and systems biology research. By uncovering novel molecular interactions, researchers can identify potential new drug targets for complex diseases, accelerating the development of effective therapies. Understanding the intricate network of protein interactions aids in identifying biomarkers for diseases, enabling more precise diagnostics and tailored treatments. Reconstructing comprehensive biological networks helps elucidate the underlying mechanisms of diseases, contributing to a systems-level understanding of biological processes. Additionally, by automating the extraction and interpretation of relevant interactions, our method reduces the manual effort required for literature reviews, allowing researchers to focus on experimental design and hypothesis testing.

Another key area for future development would be to increase the number of scientific literature sources. Currently, the ANDDigest database, used for calculating co-occurrence values and preparing text corpora for analysis, is limited to abstracts of scientific publications; incorporating information from full texts of scientific articles and patents can increase the completeness of predictions.

## Figures and Tables

**Table 1:** Evaluation of the accuracy of the fine-tuned generative language model using data from the IEPA and HPRD50 datasets.

	IEPA	HPRD50
Total Number of Examples	590	304
Positive Examples	295	152
Negative Examples	295	152
True Positives (TP)	272	141
True Negatives (TN)	272	139
False Positives (FP)	23	13
False Negatives (FN)	23	11
Sensitivity	92.2%	92.76%
Specificity	92.2%	91.45%
Precision	92.2%	91.56%
Accuracy	92.2%	92.1%
F1-score	92.2%	92.16%
MCC	0.844	0.8422

## Data Availability

The ANDSystem’s graph, used for obtaining vector representations of nodes, is available upon request. The ANDDigest is available via its web interface via the https://anddigest.sysbio.ru/ (accessed on 30 September 2024). The LLM model, fine-tuned for the protein interactions prediction task, can be accessed at the following link: https://huggingface.co/Timofey/Gemma-2-9b-it-Fused_PPI (accessed on 1 October 2024), the dataset, used for the fine-tuning of the LLM, is uploaded by the following link: https://huggingface.co/datasets/Timofey/protein_interactions_LLM_FT_dataset (accessed on 1 October 2024). The developed binary classification model, as well as all codes, examples, evaluation datasets, and results, are available at GitHub: https://github.com/ANDDigest/Text-Mining_Hybrid_RE (accessed on 21 October 2024).

## Supplementary Materials

The following supporting information can be downloaded at: https://www.mdpi.com/article/10.3390/ijms252111811/s1.

## Appendix A

**Table A1:** Distribution of edges in the initial ANDSystem graph by their types.

Edge Type	Count
catalyze	18,133,728
association	6,983,612
Regulation	1,983,853
upregulation	1,299,792
downregulation	672,405
interaction	433,046
treatment	261,807
expression regulation	185,405
proliferation regulation	86,465
expression upregulation	85,103
expression downregulation	51,332
Involvement	47,123
miRNA regulation	46,199
survival regulation	45,789
transport regulation	38,704
migration regulation	38,558
invasiveness regulation	36,408
proliferation upregulation	32,027
conversion	29,373
proliferation downregulation	28,580
survival downregulation	22,649
Expression	20,092
migration upregulation	17,968
invasiveness upregulation	16,904
activity downregulation	16,645
survival upregulation	12,286
activity upregulation	11,697
migration downregulation	11,417
invasiveness downregulation	11,357
degradation upregulation	11,273
degradation downregulation	9678
activity regulation	7585
cleavage	3917
coexpression	3352
self_renewal regulation	3026
degradation regulation	1732
self_renewal upregulation	980
self_renewal downregulation	535
catalyze modification	85
Total	30,702,487

**Table A2:** Distribution of vertices in the initial ANDSystem graph by their types.

Node Type	Count
Gene	47,244
Pathway	33,116
Metabolite	31,462
Protein	30,432
Cell	21,155
Disease	11,799
Phenotype	11,195
Component	2139
Side effect	1958
Drug	1651
miRNA	864
Total	193,015
